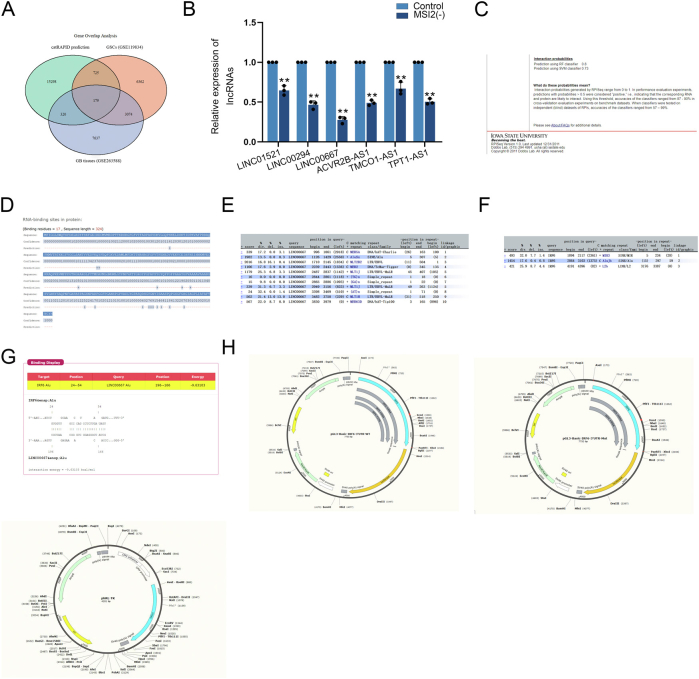# The RNA-binding protein MSI2 controls blood-tumor barrier permeability *via* LINC00667-Mediated IRF6 mRNA decay

**DOI:** 10.1016/j.jbc.2026.111208

**Published:** 2026-01-23

**Authors:** Rui Gao, Xuelei Ruan, Yixue Xue, Ping Wang, Di Wang, Tiange E, Xiaobai Liu, Libo Liu

**Affiliations:** 1 Department of Neurobiology, School of Life Sciences, China Medical University, Shenyang, China; 2 Key Laboratory of Neuro-oncology in Liaoning Province, Shenyang, China; 3 Department of Neurosurgery, Shengjing Hospital of China Medical University, Shenyang, China; 4 Neurosurgery Center, The People's Hospital of Liaoning Province, Shenyang, Liaoning, China

**Keywords:** BTB, glioblastoma, IRF6, LINC00667, MSI2, SMD

## Abstract

Increasing evidence shows that RNA-binding proteins play crucial roles in modulating the blood-tumor barrier (BTB) permeability in glioblastoma (GB). In this study, we identified elevated expression of Musashi RNA-binding protein 2 (MSI2) and Long intergenic nonprotein coding RNA 667 (LINC00667) in glioma co-cultured endothelial cells. MSI2 enhanced the stability of LINC00667, and its knockdown elevated the BTB permeability. In contrast, transcription factor interferon regulatory factor 6 (IRF6) exhibited reduced expression in glioma co-cultured endothelial cells, and its over-expression elevated the BTB permeability. Mechanistically, LINC00667 facilitated IRF6 mRNA degradation through Staufen1-mediated mRNA decay pathway. IRF6 inhibited the transcriptions of key tight junction associated proteins (ZO-1, occludin, and claudin-5) through promoter binding. That is, MSI2 knockdown down-regulated the expression of LINC00667, thereby diminishing its ability to degrade IRF6 through the Staufen1-mediated mRNA decay pathway. This led to IRF6 accumulation, which transcriptionally suppressed ZO-1, occludin and claudin-5 expression, ultimately increasing BTB permeability. Furthermore, both individual and combined modulation of MSI2 knockdown, LINC00667 knockdown and IRF6 over-expression enhanced BTB permeability to doxorubicin, thereby increasing the apoptosis rate of GB cells. Collectively, the MSI2/LINC00667/IRF6 pathway plays an important role in modulating BTB permeability, offering potential targets for new molecular therapies in GB.

Glioblastoma (GB), a World Health Organization Grade Ⅳ brain tumor, is the most common and aggressive intrinsic glioma (1, 2). Conventional therapies, such as surgery, radiotherapy and chemotherapy, have unsatisfactory therapeutic effects on it (3). Current research focuses on molecular-targeting therapy, which is a method that can interfere with specific molecules to disrupt tumor growth, invasion, and metastasis. These targeted approaches have demonstrated considerable clinical efficacy in treating multiple malignancies, such as leukemia, breast cancer, colorectal carcinoma, lung cancer, and ovarian cancer (4). However, despite advances in targeted therapies, their efficacy against GB remains limited, primarily due to the restrictive blood-tumor barrier (BTB) (5). Therefore, the urgent issue to be solved is how to open the BTB, enabling anti-tumor drugs to penetrate tumor tissue and enhance therapeutic efficacy of GB.

Increasing evidence suggests the pivotal role of RNA-binding proteins (RBPs) in modulating BTB integrity (6, 7). RBPs recognize and bind directly to single- or dsRNA to either enhance or suppress gene expression, thereby playing a crucial role in post-transcriptional gene regulation (8, 9). Musashi RNA-binding protein 2 (MSI2) exhibits aberrant expression patterns across diverse malignancies, with its dysregulation showing significant associations with clinical outcomes and disease characteristics in bladder, hepatic, pulmonary, and pancreatic cancers, as well as several leukemias (10, 11). MSI2 is up-regulated in glioma cells and significantly affects their migration, invasion, glycolipid metabolism, and proliferation (12, 13). Studies using human umbilical vein endothelial cells demonstrate that MSI2 binds to miR-301a-3p and promotes its transport to mitochondria (14). Nevertheless, the potential role of MSI2 in modulating BTB permeability remains unreported.

Long non-coding RNAs (lncRNAs), defined as RNA transcripts longer than 200 nucleotides that lack protein-coding capacity, serve as critical regulators in various cellular processes including differentiation, proliferation, apoptotic regulation, cell motility, and angiogenesis, with evidence establishing their significance in cancer pathogenesis (15). LINC00667 is located on human Chr18p11.31. Functional studies demonstrate that LINC00667 knockdown significantly inhibits the proliferative and migratory capacities of colorectal cancer cells (16). Similarly, LINC00667 silencing significantly suppresses the proliferative capacity, migratory potential, and tube formation ability in cultured non-small cell lung cancer cells (17). Notably, LASSO Cox regression analysis has identified LINC00667 as a predictive lncRNA biomarker for glioma patient prognosis (18).

Interferon regulatory factors (IRFs) are pivotal transcriptional regulators involved in modulating cell cycle progression, differentiation, programmed cell death, and antitumor immune responses (19). Notably, interferon regulatory factor 6 (IRF6) has been functionally characterized as a tumor suppressor, with its expression levels showing an inverse correlation with the aggressiveness of breast cancer (20). IRF6 induces apoptosis in glioma cells by impairing glycolysis and cell proliferation (21). Additionally, IRF6 functions as a co-repressor of peroxisome proliferator-activated receptor γ. Silencing IRF6 enhances the peroxisome proliferator-activated receptor γ -mediated protective effect on cerebrovascular endothelial cells against ischemic brain injury (22). Based on these studies, we speculate that IRF6 may play a role in endothelial cell function. However, no studies have yet reported a role for IRF6 in regulating the permeability of BTB.

Staufen1-mediated mRNA decay (SMD) represents a mammalian mRNA degradation pathway wherein the dsRNA-binding protein STAU1 recognizes dsRNA structures generated through intermolecular base-pairing interactions between Alu elements in lncRNAs and Alu elements in the 3′UTRs of target mRNAs (23). SMD is involved in the processes of adipogenesis and wound healing. Impairment of adipose tissue can promote oncogenic malignancy (24), while wound healing involves cell migration, angiogenesis, stromal remodeling, and re-epithelialization (25, 26). Notably, SMD has also been linked to the formation of angiogenic mimics in glioma (27).

This study characterized the endogenous expression levels of MSI2, LINC00667, and IRF6, and systematically examined their regulatory interplay to uncover potential mechanisms of modulating BTB permeability. These findings provide novel therapeutic targets and insights for GB treatment.

## Results

### MSI2 knockdown reduced tight junction (TJ)-associated proteins expression and elevated the permeability of BTB

**Figure 1:**
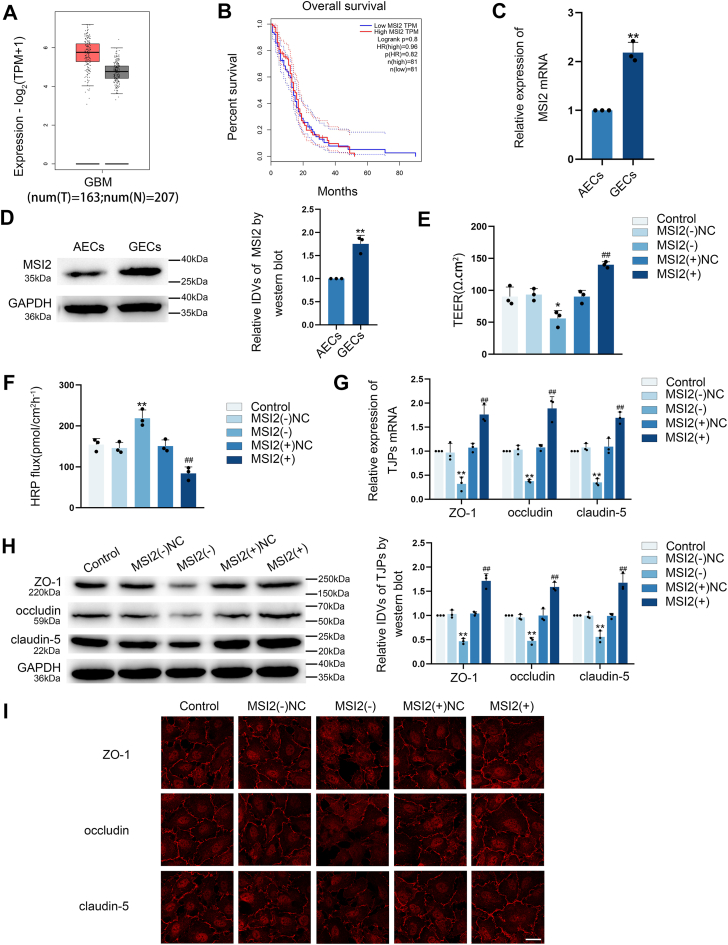
**MSI2 knockdown increased the BTB permeability through suppression of TJ-associated proteins expression.***A and B,* the expression and overall survival of MSI2 was predicted by GEPIA database. *C and D,* qRT**-**PCR and Western Blot assays demonstrated significantly elevated MSI2 mRNA and protein levels in GECs. GAPDH is used as a housekeeping gene in RT-qPCR experiments and as a loading control protein in Western blot assays. (IDVs, integrated densitometry values). Results were represented as mean ± SD (n = 3). ∗∗*p* < 0.01 *versus* AECs group. *E and F,* the effect of MSI2 on BTB permeability was assessed through TEER measurements and HRP tracer flux. Results were represented as mean ± SD (n = 3). ∗*p* < 0.05 and ∗∗*p* < 0.01 *versus* MSI2(−)NC group, **^##^***p* < 0.01 *versus* MSI2(+)NC group. *G and H,* the expression levels of ZO-1, occludin, and claudin-5 upon MSI2 modulation were examined by qRT-PCR and Western blot assays, housekeeping gene both were GAPDH. Results were represented as mean ± SD (n = 3). ∗∗*p* < 0.01 *versus* MSI2(−)NC group, **^##^***p* < 0.01 *versus* MSI2(+)NC group. *I,* IF staining was employed to examine MSI2-mediated regulation of ZO-1, occludin, and claudin-5 expression and subcellular localization in GECs. The scale bar represents 50 μm. AECs, astrocyte-co-cultured ECs; BTB, blood-tumor barrier; GEC, glioma co-cultured endothelial cell; MSI2, Musashi RNA-binding protein 2; TJ, tight junction; TEER, transendothelial electrical resistance; HRP, horseradish peroxidase; IF, Immunofluorescence.

**Supplementary Fig. S1:**
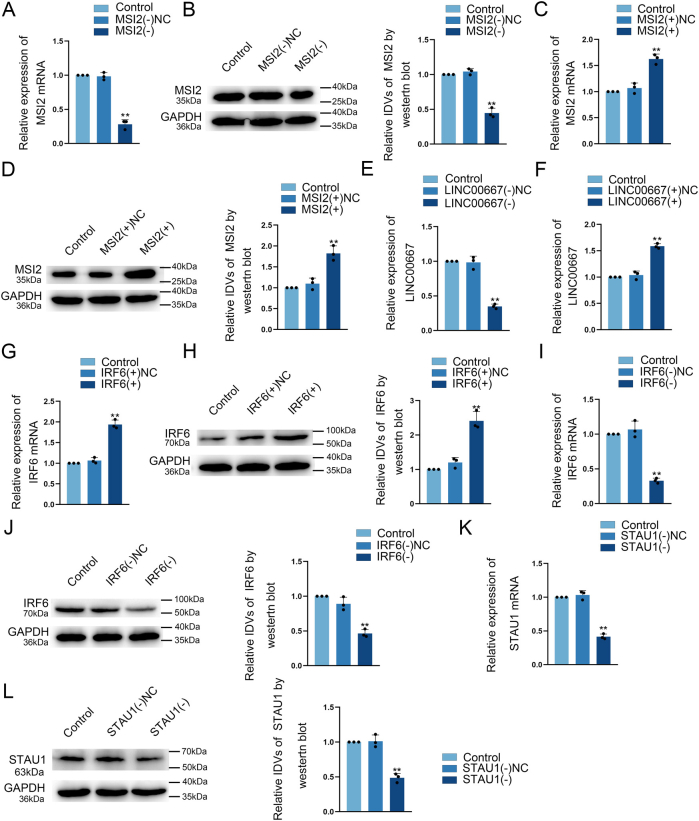


**Supplementary Fig. S2:**
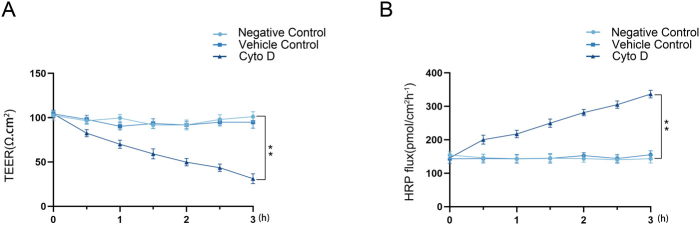


Bioinformatic analysis of the GEPIA database (http://gepia.cancer-pku.cn/) revealed elevated MSI2 expression in glioma specimens, with higher expression levels correlating with poorer patient prognosis (Fig. 1, *A* and *B*). We established blood-brain barrier (BBB) and BTB models *in vitro* using astrocyte-co-cultured ECs (AECs) and glioma co-cultured endothelial cells (GECs) to test the expression of MSI2. Quantitative analysis demonstrated that MSI2 has markedly higher expression in GECs relative to AECs (Fig. 1, *C* and D). To explore the role of MSI2 modulating BTB, ECs with MSI2 knockdown [MSI2(−)] or over-expression [MSI2(+)] were established. We established after verifying the transfection efficiency (Fig. S1, *A*–*D*). Positive control experiment of transendothelial electrical resistance (TEER) and horseradish peroxidase (HRP) were conducted to verify that these experiments could work as expected in their condition (Fig. S2, *A* and *B*). Comprehensive barrier assessment showed that MSI2 knockdown significantly disrupted BTB integrity evidenced by reduced TEER values and enhanced HRP fluxes. Conversely, MSI2 over-expression strengthened BTB function, showing increased TEER values and reduced HRP fluxes compared to the negative control (NC) of MSI2 over-expression [MSI2(+)NC] group (Fig. 1, *E* and *F*). These findings suggest that MSI2 as a key regulator of BTB permeability, demonstrating an inverse correlation between MSI2 levels and barrier integrity.

We measured ZO-1, occludin and claudin-5 levels in different groups. Both mRNA and protein levels of them were markedly lower in MSI2(−) group compared to the NC of MSI2 knockdown [MSI2(−)NC] group, while the MSI2(+) group exhibited higher levels than its in MSI2(+)NC group (Fig. 1, *G* and *H*). IF assay further confirmed these finding, demonstrating continuous membrane localization of tight junction (TJ)-associated proteins in GECs from Control and NC groups. Compared with the NC groups, the MSI2(−) group showed discontinuous distribution and reduced expression of these proteins, whereas the MSI2(+) group maintained continuous membrane localization with enhanced expression (Fig. 1*I*). These findings demonstrate that MSI2 knockdown elevates BTB permeability through suppression of TJ-associated proteins expression.

### MSI2 bound and increased the stability of LINC00667

**Figure 2:**
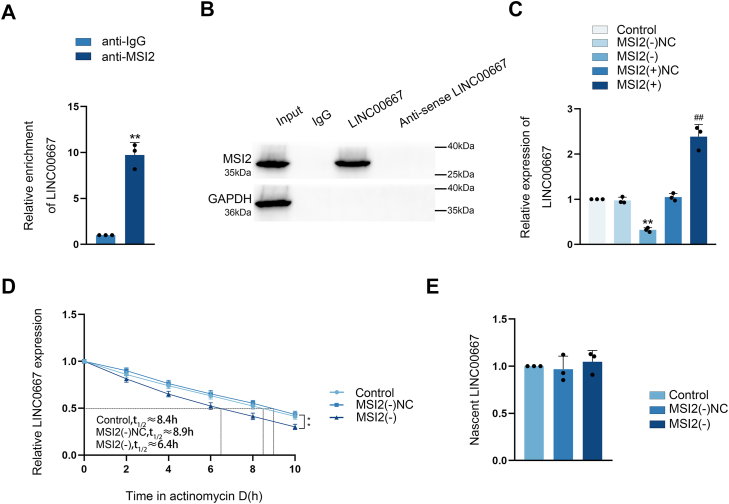
**MSI2 bound and stabilized the expression of LINC00667.***A,* the interaction between MSI2 and LINC00667 was analyzed by RIP, with LINC00667 enrichment quantified by qRT-PCR. Results were represented as mean ± SD (n = 3). ∗∗*p* < 0.01 *versus* anti-IgG group. *B,* the presence of MSI2 and GAPDH in LINC00667-containing ribonucleoprotein complexes, purified *via* RNA pull-down assay, was verified by Western blot. *C,* the influence of MSI2 on LINC00667 expression levels in GECs was evaluated by qRT-PCR assay, housekeeping gene was GAPDH. Data were represented as mean ± SD (n = 3). ∗∗*p* < 0.01 *versus* MSI2(−)NC group, ^##^*p* < 0.01 *versus* MSI2(+)NC group. *D,* LINC00667 mRNA stability in GECs following MSI2 knockdown was evaluated using actinomycin D-mediated transcriptional inhibition assay. Data were represented as mean ± SD (n = 3). *E,* nascent RNA capture assay was employed to assess MSI2 knockdown effects on newly synthesized LINC00667 transcripts in GECs. GEC, glioma co-cultured endothelial cell; MSI2, Musashi RNA-binding protein 2; LINC00667, long intergenic nonprotein coding RNA 667; IF, Immunofluorescence.

To identify the key downstream RNA targets through which MSI2 exerts its oncogenic function, transcriptome profiling revealed To investigate potential interactions between MSI2 and LINC00667, the RPISeq database (http://pridb.gdcb.iastate.edu/RPISeq/index.html) and RNAInter (http://rnainter.org/IntaRNA/) were used. Both platforms consistently predicted a binding interaction, as shown in Figure S4, *C* and *D*. Meanwhile, RIP experiment revealed a significant enrichment of LINC00667 in anti-MSI2 group relative to the anti-IgG group (Fig. 2*A*). RNA pull-down (RPD) experiment revealed a target binding interaction between MSI2 and LINC00667 (Fig. 2*B*). These findings suggested that MSI2 is the binding protein for LINC00667. To explore how MSI2 regulates LINC00667, we assessed LINC00667 expression levels *via* quantitative real-time PCR (qRT-PCR) assay following MSI2 knockdown and over-expression. MSI2 knockdown markedly reduced LINC00667 expression, whereas MSI2 over-expression increased it (Fig. 2*C*). Furthermore, we determined the impact of MSI2 on the LINC00667 RNA stability using actinomycin D treatment and analyzed nascent transcript production *via* RNA capture assays. MSI2 shortened the half-life of LINC00667 from 8.9 h to 6.4 h without altering nascent RNA level (Fig. 2, *D* and *E*), suggesting that MSI2 post-transcriptionally regulates LINC00667 through binding-mediated stabilization.

### LINC00667 knockdown down-regulated TJ-associated proteins expressions and elevated the permeability of BTB

**Figure 3:**
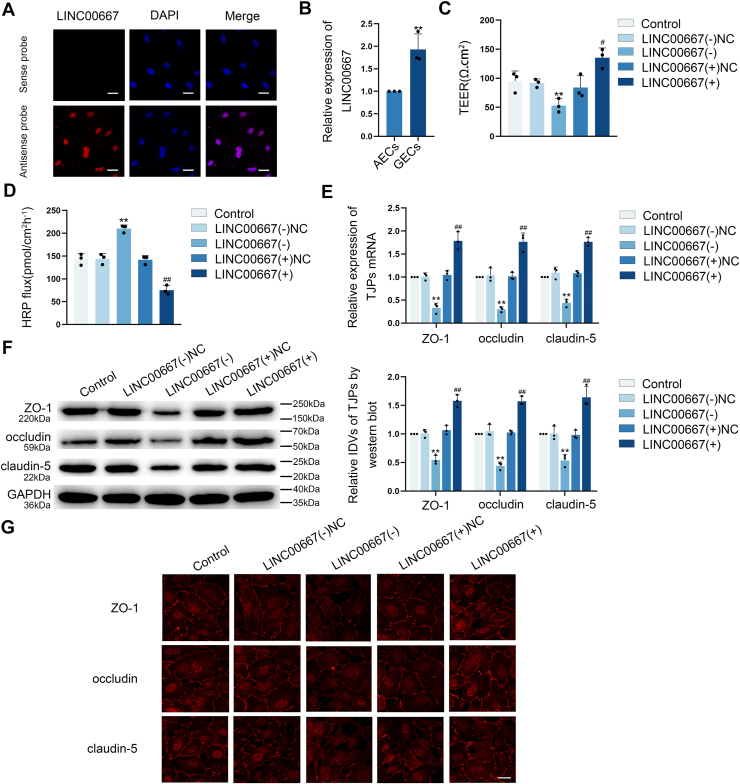
**LINC00667 knockdown increased the BTB permeability through suppression of TJ-associated proteins expression.***A,* the expression pattern and subcellular distribution of LINC00667 in GECs were visualized using FISH (*red*, LINC00667; *blue*, DAPI nuclear staining). The scale bar represents 50 μm. *B,* LINC00667 mRNA level was quantified by qRT-PCR assay, housekeeping gene was GAPDH. Data were represented as mean ± SD (n = 3). ∗∗*p* < 0.01 *versus* AECs group. *C and D*, the effect of LINC00667 on BTB permeability was assessed through TEER measurements and HRP tracer flux. Data were represented as mean ± SD (n = 3). ∗∗*p* < 0.01 *versus* LINC00667 konckdown NC [LINC00667(−)NC] group, ^#^*p* < 0.05 and ^##^*p* < 0.01 *versus* LINC00667 over-expression NC [LINC00667(+)NC] group. *E and F,* the expression levels of ZO-1, occludin, and claudin-5 upon LINC00667 modulation were examined by qRT-PCR and Western blot assays, housekeeping gene both were GAPDH. Data were represented as mean ± SD (n = 3). ∗∗*p* < 0.01 *versus* LINC00667(−)NC group, ^##^*p* < 0.01 *versus* LINC00667(+)NC group. *G,* IF staining was employed to examine LINC00667-mediated regulation of ZO-1, occludin, and claudin-5 expression and subcellular localization in GECs. ThE scale bar represents 50 μm. AECs, astrocyte-co-cultured ECs; BTB, blood-tumor barrier; GEC, glioma co-cultured endothelial cell; LINC00667, long intergenic nonprotein coding RNA 667; TJ, tight junction; TEER, transendothelial electrical resistance; HRP, horseradish peroxidase; IF, Immunofluorescence; NC, negative control.

FISH assay demonstrated predominant nuclear localization of LINC00667 in GECs (Fig. 3*A*). qRT-PCR assay revealed significantly elevated LINC00667 expression in GECs relative to AECs (Fig. 3*B*). To confirm the function of LINC00667 adjusting the BTB permeability, LINC00667 knockdown [LINC00667(−)] and over-expressed [LINC00667(+)] BTB models *in vitro* were established (Fig. S1, *E* and *F*). As shown in Figure 3, *C* and *D*, LINC00667 knockdown markedly reduced TEER values while enhancing HRP flux compared to the NC. Conversely, LINC00667 over-expression elevated TEER values and reduced HRP flux relative to the NC [LINC00667(+)NC] group. These findings demonstrated that LINC00667 knockdown leads to increased BTB permeability.

In addition, qRT-PCR and Western blot analyses revealed that LINC00667 knockdown substantially decreased ZO-1, occludin, and claudin-5 expression at both transcriptional and translational levels, whereas LINC00667 over-expression enhanced their expression compared to respective controls (Fig. 3, *E* and *F*). IF assay demonstrated continuous membrane localization of ZO-1, occludin, and claudin-5 in control and NC groups. However, LINC00667 knockdown disrupted this membrane distribution pattern and reduced expression levels, while LINC00667 over-expression enhanced TJ-associated proteins localization and expression (Fig. 3*G*). Collectively, these data indicate that LINC00667 knockdown enhances BTB permeability through suppression of TJ-associated proteins expression.

### MSI2 knockdown elevated the permeability through modulation of LINC00667 RNA stability

**Figure 4:**
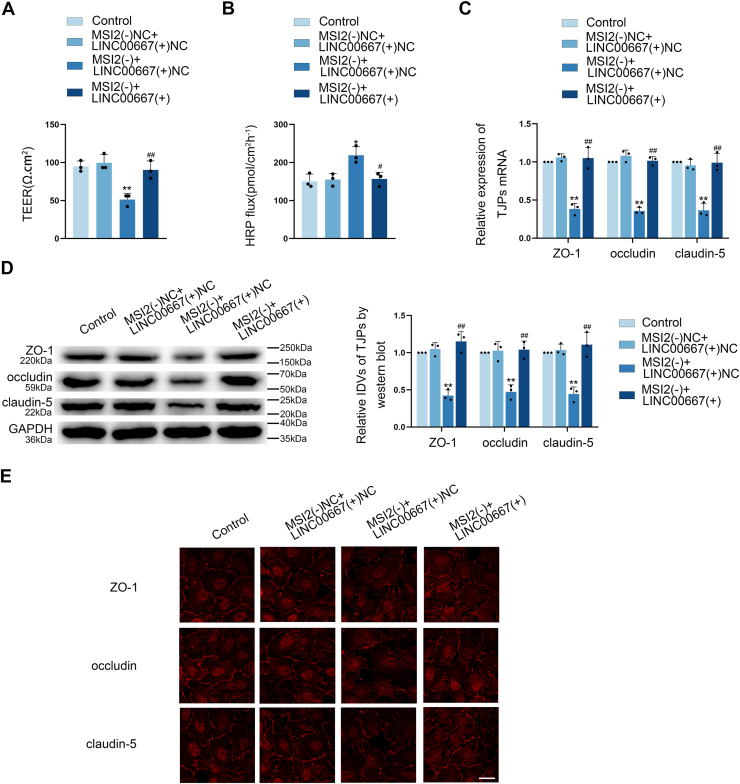
**MSI2 knockdown enhanced BTB permeability by reducing LINC00667 stability.***A and B,* the impact of LINC00667 over-expression on BTB permeability was assessed through TEER measurements and HRP tracer flux in MSI2-knockdown cells. Data were represented as mean ± SD (n = 3). ∗*p* < 0.05 and ∗∗*p* < 0.01 *versus* MSI2(−)NC + LINC00667(+)NC group, ^#^*p* < 0.05 and ^##^*p* < 0.01 *versus* MSI2(−) + LINC00667(+)NC group. *C and D,* the impact of LINC00667 over-expression on TJ-associated proteins expression was analyzed through qRT-PCR and Western blot assays in MSI2-knockdown cells, housekeeping gene both were GAPDH. Data were represented as mean ± SD (n = 3). ∗∗*p* < 0.01 *versus* MSI2(−)NC + LINC00667(+)NC group, ^##^*p* < 0.01 *versus* MSI2(−) + LINC00667(+)NC group. *E,* IF assay was conducted to evaluate changes in the expression and subcellular localization of TJ-associated proteins resulting from LINC00667 over-expression in MSI2-knockdown cells. The scale bar represents 50 μm. BTB, blood-tumor barrier; MSI2, Musashi RNA-binding protein 2; LINC00667, long intergenic nonprotein coding RNA 667; TJ, tight junction; TEER, transendothelial electrical resistance; HRP, horseradish peroxidase.

To investigate MSI2-mediated regulation of BTB permeability through LINC00667, the BTB model *in vitro* combining MSI2 knockdown with LINC00667 over-expression was established. Rescue experiments showed that LINC00667 over-expression reversed the barrier-disrupting effects of MSI2 knockdown, including decreased TEER values (Fig. 4*A*), increased HRP flux (Fig. 4*B*), and down-regulated expression of TJ-associated proteins (Fig. 4, *C*–*E*). These findings demonstrate that MSI2 regulates BTB permeability by controlling LINC00667 stability.

### IRF6 over-expression down-regulated TJ-associated proteins expression and elevated the BTB permeability

**Figure 5:**
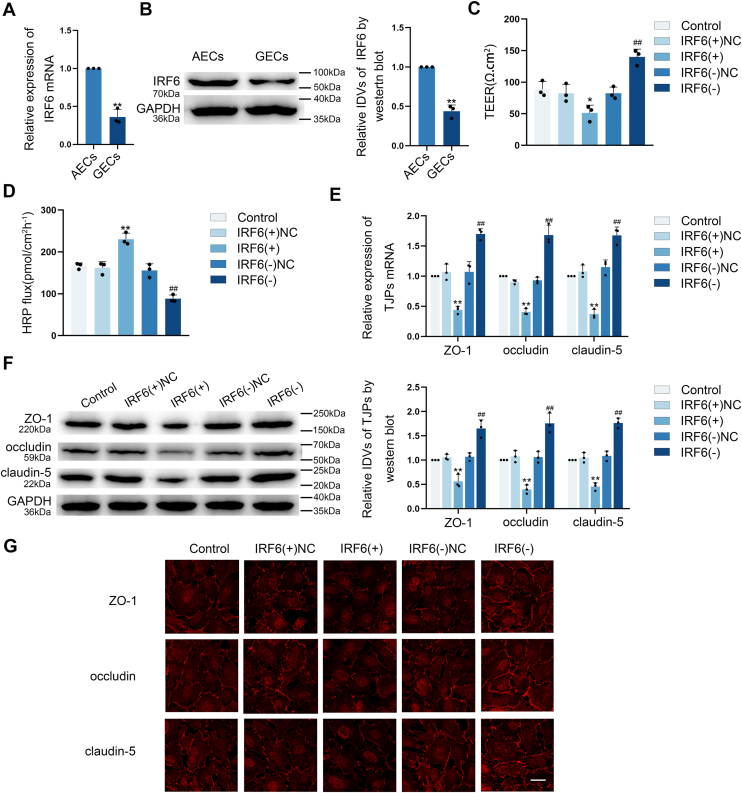
**IRF6 over-expression enhanced BTB permeability through suppression of TJ-associated proteins expression.***A and B,* both qRT**-**PCR and Western Blot assays demonstrated significantly reduced IRF6 mRNA and protein levels in GECs, housekeeping gene both were GAPDH. Data were represented as mean ± SD (n = 3). ∗∗*p* < 0.01 *versus* AECs group. *C and D,* the contribution of IRF6 to BTB regulation was assessed through TEER measurements and HRP tracer flux. Data were represented as mean ± SD (n = 3). ∗*p* < 0.05 and ∗∗*p* < 0.01 *versus* IRF6 over-expression NC [IRF6(+)NC] group, ^##^*p* < 0.01 *versus* IRF6 knockdown NC [IRF6(−)NC] group. *E and F,* the regulatory effect of IRF6 on ZO-1, occludin and claudin-5 expression in GECs was assessed by qRT-PCR and Western blot assays, housekeeping gene both were GAPDH. Data were represented as mean ± SD (n = 3). ∗∗*p* < 0.01 *versus* IRF6(+) NC group, ^##^*p* < 0.01 *versus* IRF6(−)NC group. *G,* IF staining was employed to examine IRF6-mediated regulation of ZO-1, occludin, and claudin-5 expression and subcellular localization in GECs. The scale bar represents 50 μm. AECs, astrocyte-co-cultured ECs; BTB, blood-tumor barrier; GEC, glioma co-cultured endothelial cell; LINC00667, long intergenic nonprotein coding RNA 667; IRF6, interferon regulatory factor 6; TJ, tight junction; TEER, transendothelial electrical resistance; HRP, horseradish peroxidase; IF, Immunofluorescence.

Comparative analysis showed significantly lower IRF6 expression in GECs relative to AECs at both transcriptional and translational levels (Fig. 5, *A* and *B*). To functionally characterize IRF6 in BTB regulation, the BTB models *in vitro* with either IRF6 knockdown [IRF6(−)] or over-expression [IRF6(+)] were established, respectively (Fig. S1, *G*–*J*). TEER measurement and HRP flux assays demonstrated that IRF6 over-expression significantly decreased TEER values while increasing HRP permeability compared to NC [IRF6(+)NC] groups. Conversely, IRF6 knockdown showed opposite changes (Fig. 5, *C* and *D*). These findings suggested that IRF6 over-expression significantly elevated the BTB permeability.

In addition, qRT-PCR and Western blot analyses revealed that IRF6 over-expression substantially decreased ZO-1, occludin, and claudin-5 expression at both transcriptional and translational levels, whereas IRF6 knockdown enhanced their expression compared to respective controls (Fig. 5, *E* and *F*). IF assay demonstrated continuous membrane localization of ZO-1, occludin, and claudin-5 in control and NC groups. However, IRF6 over-expression disrupted this membrane distribution pattern and reduced expression levels, while IRF6 knockdown enhanced TJ-associated proteins localization and expression (Fig. 5*G*). Collectively, these data indicate that IRF6 over-expression enhances BTB permeability through suppression of TJ-associated proteins expression.

### Evidence for LINC00667 modulated the BTB permeability by promoting STAU1-mediated decay of IRF6 mRNA

**Figure 6:**
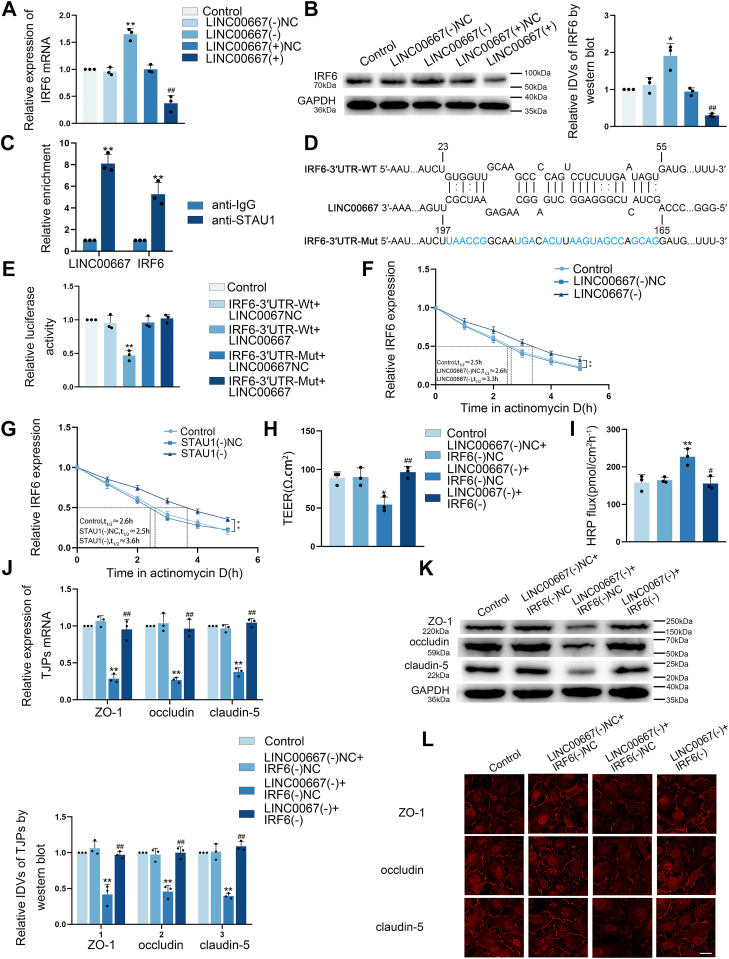
**LINC00667 degraded IRF6 mRNA by** Staufen1-mediated mRNA decay **pathway to regulate BTB permeability.***A and B,* the impact of LINC00667 on IRF6 expression in GECs was evaluated through qRT-PCR and Western blot assays, housekeeping gene both were GAPDH. Data were represented as mean ± SD (n = 3). ∗*p* < 0.05 and ∗∗*p* < 0.01 *versus* LINC00667(−)NC group, ^##^*p* < 0.01 *versus* LINC00667(+)NC group. *C,* RIP assay was employed to characterize the specific interactions of STAU1 with both LINC00667 and IRF6. Data were represented as mean ± SD (n = 3). ∗∗*p* < 0.01 *versus* anti-IgG group. *D,* the WT binding site for LINC00667 in the IRF6 3′UTR (IRF6-3′UTR-WT) and its mutant variant (IRF6-3′UTR-Mut) were both characterized. *E,* dual-luciferase reporter gene assay was utilized to verify the molecular interaction between LINC00667 and IRF6. Data were represented as mean ± SD (n = 3). ∗∗*p* < 0.01 *versus* IRF6-3′UTR-WT + LINC00667NC group. *F and G,* the percentage remaining of IRF6 mRNA at various time points following actinomycin D treatment in Control, LINC00667(−)NC, and LINC00667(−) groups. The percentage remaining of IRF6 mRNA at various time points following actinomycin D treatment in Control, STAU1(−)NC, and STAU1(−) groups. *H and I,* BTB permeability was assessed through TEER measurements and HRP tracer flux. Data were represented as mean ± SD (n = 3). ∗*p* < 0.05 and ∗∗*p* < 0.01 *versus* LINC00667(−)NC + IRF6(−)NC group, ^#^*p* < 0.05 and ^##^*p* < 0.01 *versus* LINC00667(−) + IRF6(−)NC group. *J and K,* the expression of TJ-associated proteins was analyzed using qRT-PCR and Western blot assays, housekeeping gene both were GAPDH. Data were represented as mean ± SD (n = 3). ∗∗*p* < 0.01 *versus* LINC00667(−)NC + IRF6(−)NC group, ^##^*p* < 0.01 *versus* LINC00667(−) + IRF6(−)NC group. *L,* IF staining was employed to examine the expression and subcellular localization of ZO-1, occludin, and claudin-5 in GECs. Scale bar represents 50 μm. BTB, blood-tumor barrier; GEC, glioma co-cultured endothelial cell; LINC00667, long intergenic nonprotein coding RNA 667; IRF6, interferon regulatory factor 6; TJ, tight junction; TEER, transendothelial electrical resistance; HRP, horseradish peroxidase; IF, Immunofluorescence.

Notably, qRT-PCR and Western blot analyses demonstrated that IRF6 mRNA and protein levels were reduced upon LINC00667 over-expression but elevated following LINC00667 knockdown (Fig. 6, *A* and *B*). To investigate how LINC00667 modulates IRF6 expression, we analyzed the RepeatMasker database (http://repeatmasker.org/cgi-bin/WEBRepeatMasker), which revealed conserved Alu elements in both LINC00667 and the 3′UTR of IRF6 mRNA. RNAInter (http://rnainter.org/IntaRNA/) predicted that LINC00667 and IRF6 mRNA 3′UTR region complementarily paired by Alu sequences (Fig. S4, *E*–*G*). The above analysis suggested that LINC00667 might degrade IRF6 mRNA *via* the SMD pathway. Given that SMD mechanism involving STAU1 recognizing and binding of dsRNA formed through Alu element pairing between lncRNAs and target mRNAs, we performed RIP assays to examine potential interactions among LINC00667, IRF6, and STAU1. The data demonstrated significant enrichment of both LINC00667 and IRF6 in anti-STAU1 immunoprecipitates compared to IgG controls (Fig. 6*C*). To functionally validate the LINC00667-IRF6 3′UTR interaction, we conducted dual-luciferase reporter assays. The results revealed WT IRF6 3′UTR showed significantly reduced relative luciferase activity when co-expressed with LINC00667 compared to NCs. This suppressive effect was abolished when using mutant IRF6 3′UTR constructs (Fig. 6, *D* and *E*). Meanwhile, the actinomycin D assay revealed that the half-life of IRF6 increased from 2.5 h to 3.3 h after LINC00667 knockdown (Fig. 6*F*). These findings supported the hypothesis that LINC00667 binds to IRF6 mRNA 3′UTRs through Alu sequence, thereby promoting IRF6 degradation. To provide evidence that the involvement of the SMD pathway in LINC00667-mediated IRF6 degradation by establishing STAU1-knockdown [STAU1(−)] ECs. Following validation of knockdown efficiency (Fig. S1, *K* and *L*), actinomycin D assay showed that STAU1 knockdown extended the half-life of IRF6 mRNA from 2.6 h to 3.6 h (Fig. 6*G*). These results suggest that LINC00667 over-expression degrades IRF6 mRNA through the SMD pathway.

To verify IRF6's role in LINC00667-mediated BTB regulation, we generated the BTB model *in vitro* with combined LINC00667 and IRF6 knockdown. Strikingly, IRF6 knockdown reversed the barrier-disruptive effects of LINC00667 knockdown, including decreased TEER values, increased HRP fluxes, and down-regulation of TJ-associated proteins (Fig. 6, *H*–*L*). The above results demonstrate that LINC00667 knockdown elevates IRF6 expression by preventing IRF6 mRNA degradation, thereby increasing the BTB permeability.

### IRF6 interacted with the promoter regions of TJ-associated proteins and repressed their expression at the transcriptional level

**Figure 7:**
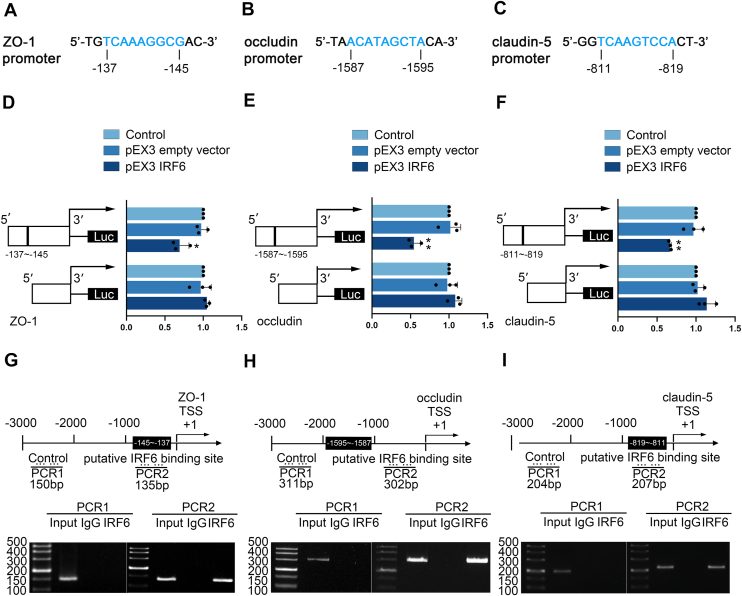
**IRF6 directly bound the promoter sequences of ZO-1, occludin, and claudin-5, inhibiting their transcriptional activity.***A–C,* bioinformatic analysis identified putative IRF6-binding motifs in the promoter sequences of ZO-1, occludin, and claudin-5. *D–F,* dual-luciferase reporter assay was employed to evaluate IRF6-mediated modulation of ZO-1, occludin, and claudin-5 promoter activities. Data were represented as mean ± SD (n = 3, each). ∗∗*p* < 0.01 *versus* pEX3 empty vector group. *G–I*, chromatin immunoprecipitation experiment was conducted to validate IRF6 binding to the promoter regions (positioned at the −3000 bp) of ZO-1, occludin, and claudin-5 (relative to their TSSs). The experimental design included NC amplifications (PCR1) and specific detection of predicted IRF6-binding sites (PCR2). IRF6, interferon regulatory factor 6.

JASPAR database analysis (https://jaspar.genereg.net/) was used to analyze and identify putative IRF6 binding sites in the promoters of ZO-1, occludin, and claudin-5 (Fig. 7, *A*–*C*). Subsequent dual-luciferase assay demonstrated that IRF6 (pEX3-IRF6) significantly suppressed the promoter activity of these TJ-associated proteins compared to the pEX3 empty vector. Notably, this repression was abolished when the predicted IRF6 binding sites were deleted, with promoter activities showing no significant differences between the experimental and empty vector groups (Fig. 7, *D*–*F*). Chromatin immunoprecipitation (ChIP) experiment confirmed specific enrichment of IRF6 at the predicted binding sites within ZO-1, occludin, and claudin-5 promoters, while control regions showed no significant binding (Fig. 7, *G*–*I*). The above findings demonstrate that IRF6 transcriptionally represses TJ-associated proteins expression through promoter-specific interactions.

### Targeted modulation of MSI2 knockdown, LINC00667 knockdown and IRF6 over-expression enhanced doxorubicin (Dox) delivery accross the BTB to trigger GB cell apoptosis

**Figure 8:**
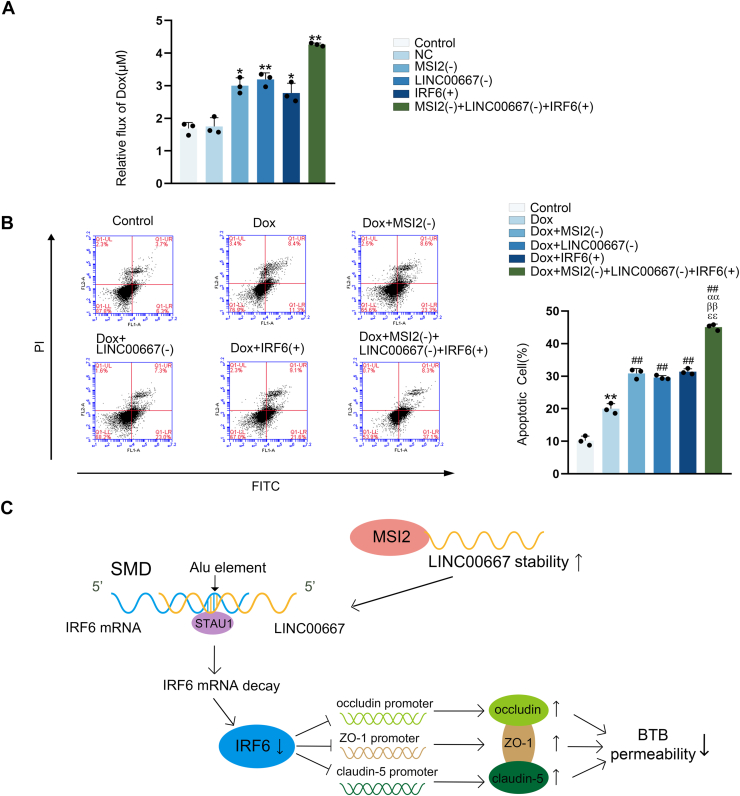
**The individual or combined application of MSI2 knockdown, LINC00667 knockdown, and IRF6 over-expression promoted Dox penetration through the BTB, subsequently inducing** glioblastoma **cell apoptosis.***A,* dox penetration through the BTB model *in vitro* was quantified by spectrophotometer. Data were represented as mean ± SD (n = 3, each).∗*p* < 0.05 and ∗∗*p* < 0.01 *versus* NC group. *B,* the percentage of apoptotic U251 cells was measured using flow cytometry. Data were represented as mean ± SD (n = 3). ∗*p* < 0.05 and ∗∗*p* < 0.01 *versus* Control group, ^##^*p* < 0.01 *versus* Dox group, ^αα^*P* < 0.01 *versus* Dox + MSI2(−), ^ββ^*P* < 0.01 *versus* Dox + LINC00667(−), ^ℇℇ^*P* < 0.01 *versus* Dox + IRF6(+). Q1-lower left: Annexin V-/PI-, Q1-lower right: Annexin V+/PI-, Q1-upper right: Annexin V+/PI+, Q1-UL: Annexin V-/PI+. Apoptotic cell (%) = Q1-lower right + Q1-uper right. *C,* working model illustrating the proposed mechanism by which the MSI2/LINC00667/IRF6 signaling cascade regulates BTB integrity through the Staufen1-mediated mRNA decay pathway. BTB, blood-tumor barrier; MSI2, Musashi RNA-binding protein 2; LINC00667, long intergenic nonprotein coding RNA 667; IRF6, interferon regulatory factor 6; Dox, doxorubicin

**Supplementary Fig. S3:**
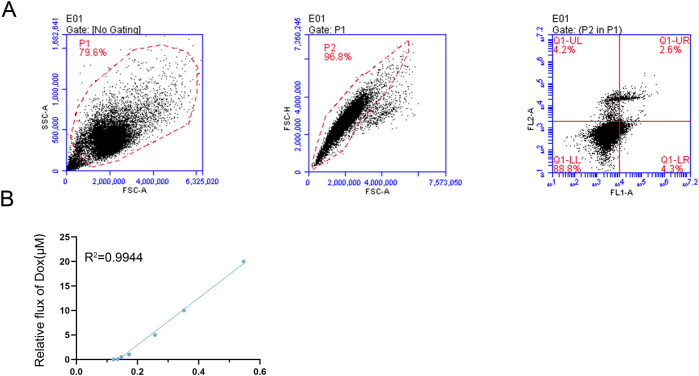


We established the BTB models *in vitro* with individual or combined genetic modifications of MSI2, LINC00667, and IRF6 to evaluate their impact on doxorubicin (Dox) penetration and subsequent GB cell apoptosis. First, we established the standard curve of Dox (Fig. S3*B*). Then, Figure 8*A* demonstrated significantly enhanced Dox flux across the BTB in MSI2(−), LINC00667(−), IRF6(+), and MSI2(−) + LINC00667(−) + IRF6(+) groups compared to the NC group, with the most pronounced increase observed in the MSI2(−) + LINC00667(−) + IRF6(+) group. Meanwhile, Figure 8*B* showed that all Dox-treated groups exhibited increased apoptosis rates, with the greatest response observed in the Dox + MSI2(−) + LINC00667(−) + IRF6(+) group. These results demonstrated that both individual and combined modulation of MSI2 knockdown, LINC00667 knockdown, and IRF6 over-expression enhanced the BTB permeability to Dox, thereby enhancing the apoptosis rate of GB cells. Figure 8*C* illustrated the proposed mechanism through which the MSI2/LINC00667/IRF6 modulated the BTB permeability by activating the SMD pathway.

## Discussion

The BBB serves as both a physical and physiological separation that regulates exchange between peripheral circulation and the central nervous system, thereby preserving neuronal environmental stability (28, 29). Meanwhile, the BBB and BTB also present significant challenges for drug delivery in central nervous system disorders, including Parkinson's disease and brain tumors, by restricting therapeutic agents' penetration into target tissues (30). Chemotherapeutic agents cross the BBB and BTB primarily through two primary mechanisms: the transcellular (transmembrane) pathway and the paracellular (intercellular) pathway (31, 32).

Given the BBB's limited vesicular transport capacity (33), along with the known impact of TJ-associated proteins expression changes on BBB integrity and the molecular penetration (34, 35, 36), modulating the paracellular pathway may offer an effective strategy to increase BTB permeability, thereby improving drug delivery and therapeutic efficacy in GB. This study revealed that MSI2, which was abundantly expressed in GECs, interacted with and stabilized LINC00667. Subsequently, LINC00667 promoted IRF6 mRNA degradation *via* activation of the SMD pathway. IRF6, in turn, transcriptionally suppressed the expression of TJ-associated proteins, consequently modulating the BTB permeability. Both individual and combined modulation of MSI2 knockdown, LINC00667 knockdown, and IRF6 over-expression effectively enhanced the permeability of Dox across the BTB, inducing apoptosis in GB cells.

RBPs interact with RNA to form ribonucleoproteins (RNPs) complexes, which regulate critical post-transcriptional mechanisms in gene expression regulation (8). By binding target mRNAs and modulating RNA stability and translational efficiency, RBPs can significantly influence cancer occurrence, malignant progression, and drug resistance. This is achieved through the altered expression of key oncogenic regulators including HuR, MSI1, and MSI2 (37). The study demonstrated elevated expression of MSI2 in GB tissues, with its knockdown markedly suppressing tumor cell proliferation, migration, and invasive capacity (13). Additionally, the study revealed high MSI2 expression in human umbilical vein endothelial cells, where it interacts with miR-301a-3p to facilitate mitochondrial translocation, thereby modulating endothelial cell function (14). Our study demonstrated elevated MSI2 expression in GECs, aligning with previous reports and supporting its role in modulating GB endothelial function. However, the potential involvement of MSI2 in BTB permeability regulation remained unknown. Our findings revealed that MSI2 knockdown significantly reduced the expression of TJ-associated proteins and enhanced BTB permeability *via* the paracellular pathway. The precise molecular mechanisms underlying this regulation warrant further exploration.

LncRNAs serve as fundamental regulators involved in various physiological and pathological processes. They participate in diverse cellular processes by functioning as signaling molecules, molecular decoys, structural scaffolds, regulatory guides, enhancer RNAs, or short peptide-coding transcripts (15, 38). RBPs, as important regulators of lncRNAs in cancer, modulatie lncRNA stability to mediate various biological oncogenic functions (39). Research has shown that HuR binds and stabilizes lncRNA-HGBC, facilitating the growth and metastatic potential of gallbladder cancer cells (37). Similarly, FXR1 is up-regulated in both glioma tissues and cellular models, where it modulates glioma cell behavior through stabilizing MIR17HG (40). To identify lncRNAs potentially involved in MSI2-mediated regulation of BTB permeability, our group initially used the catRAPID database to identify MSI2-binding lncRNAs, while simultaneously mining the GEO project for lncRNAs upregulated in GB tissues (GSE263588) and glioma stem cells (GSCs, GSE119834). Integrated analysis of these three datasets yielded 179 lncRNAs that were highly expressed in GB tissues, GSCs and could bind MSI2 (Fig. S4*A*). Building upon the RepeatMasker analysis of Alu elements in these lncRNAs, we selected six lncRNAs (TPT1-AS1, LINC01521, LINC00294, ACVR2B-AS1, TMCO1-AS1, LINC00667) of interest with relatively high predicted MSI2-binding affinity for verification. Upon MSI2 knockdown, all six lncRNAs showed decreased expression, with LINC00667 being one of the most significantly downregulated (Fig. S4*B*). Additionally, interaction predictions using RPISeq (http://pridb.gdcb.iastate.edu/RPISeq/index.html) and RNAInter (http://rnainter.org/IntaRNA/) software supported a binding relationship between MSI2 and LINC00667 (Fig. S4, *C* and *D*). Consequently, we selected LINC00667 as a downstream effector of MSI2 for further investigation. Our findings revealed that MSI2 knockdown down-regulated LINC00667 expression, while MSI2 over-expression up-regulated it. RIP and RPD assays demonstrated that MSI2 bound to LINC00667. Furthermore, our experimental data showed that MSI2 knockdown did not change LINC00667 transcription rates but reduced its RNA stability by decreasing transcript half-life. These findings demonstrate that MSI2 interacts with and stabilizes LINC00667, consistent with previous observations reported by Hu *et al.* (37).

Emerging evidence discovers that LINC00667 exhibits oncogenic properties across multiple cancer types, highlighting its dual potential as both a biomarker and therapeutic target (41). In hepatocellular carcinoma, elevated LINC00667 expression correlates with poor clinical outcomes. Similarly, in colorectal cancer cells, it promotes tumor progression by enhancing proliferative capacity and metastatic potential (16, 42). However, it remains unknown whether LINC00667 can affect the endothelial function in GB. To investigate the expression and functional role of LINC00667 in GECs and its influence on BTB permeability, FISH and qRT-PCR assyas were conducted. The results found that LINC00667 was localized in the nucleus and exhibited high expression levelsin GECs, suggesting its potential role in regulating endothelial function. Additionally, this study revealed that LINC00667 knockdown could enhance BTB permeability by reducing the expressions of TJ-assocaited proteins. To further verify whether MSI2 regulates the BTB permeability by affecting the stability of LINC00667, GECs were co-transfected with MSI2 knockdown and LINC00667 over-expressed plasmids. The results showed LINC00667 over-expression rescued the MSI2 knockdown-induced effects, including decreased TEER values, increased HRP fluxes, and reduced expression of TJ-associated proteins. These findings demonstrated that MSI2 knockdown could down-regulate the expression of TJ-associated proteins by destabilizing LINC00667, consequently enhancing BTB permeability. Further studies are needed to uncover the exact mechanisms by which LINC00667 controls the expression of TJ-assocaited proteins.

LncRNAs regulate the function of downstream genes through various mechanisms, including the competitive endogenous RNA pathway (43), serving as scaffolds for regulating protein-protein interactions (44), and the SMD pathway, which is dependent on translation and ubiquitously exist in mammalian cells (45). In particular, the SMD pathway has garnered increasing attention. For instance, LINC00662 regulates the BTB permeability by influencing the stability of KLF4 mRNA *via* the SMD pathway (46). However, it remains unclear whether LINC00667 regulates the stability of downstream gene mRNA through the SMD pathway, thereby modulating BTB permeability. Previous reports indicate that IRF6 play a critical role in regulating endothelial cell maturation and integrity (22, 47). IRF6 over-expression is known to damage endothelial integrity (22). These findings suggested a potential inverse correlation between LINC00667 and IRF6 in the regulation of barrier integrity. Indeed, our study showed that LINC00667 knockdown significantly up-regulated the expression of IRF6, while LINC00667 over-expression had the opposite effect. Simultaneously, potential sequence complementarity between the Alu element in LINC00667 and the 3′UTR of IRF6 mRNA was predicted by using bioinformatic software RepeatMasker and RNAInter. This interaction was experimentally confirmed by dual-luciferase assay, demonstrating Alu-mediated binding. Furthermore, RIP experiments demonstrated that both LINC00667 and IRF6 could bind to STAU1. The actinomycin D experiment revealed that LINC00667 knockdown significantly increased IRF6 mRNA stability, suggesting LINC00667 promotes IRF6 degradation through the SMD pathway. This finding aligns with previous reports showing that SNHG14 similarly regulates IRF6 through SMD (21), collectively indicating SMD as a critical post-transcriptional regulatory mechanism for lncRNAs-mediated IRF6 modulation.

IRFs belong to a family of transcription factors, with nine members (IRF1∼IRF9) currently identified in mammals (48). IRF6 plays a crucial role in epidermal differentiation and barrier function (49). Its expression pattern is particularly interesting, as it disappears during the transition from normal mammary epithelium to invasive breast cancer (21). Similarly, in this study, GECs exhibited minimal endogenous IRF6 expression. IRF6 Over-expression disrupted the BTB *via* reduced expression of TJ-associated proteins. Mechanistic investigations using dual-luciferase reporter assays and ChIP demonstrated direct binding of IRF6 to promoter regions of ZO-1, occludin, and claudin-5 genes. These results indicated that IRF6 transcriptionally repressed the expression of TJ-associated proteins. This finding is consistent with the results of Lu *et al.* (21), who found that IRF6 blocks PKM2 and GLUT1 transcription, thereby reducing glycolysis, decreasing cell proliferation, and inducing apoptosis in GB cells.

Dox is widely applied in clinical therapy of many cancers, including GB, due to its good efficacy in anti-cancer. However, the BTB significantly limits its therapeutic efficacy by preventing adequate drug accumulation within tumor tissues (50). Our study demonstrated that individual modulation through MSI2 knockdown, LINC00667 knockdown, or IRF6 over-expression enhanced Dox permeability across the BTB, with the triple-combination strategy showing maximal efficacy. Apoptosis assays further revealed that this combinatorial approach most effectively promoted Dox-induced apoptosis in GB cells, demonstrating both improved drug penetration and enhanced therapeutic efficacy.

Our study revealed distinct expression patterns in GECs, with elevated MSI2 and LINC00667 levels but diminished IRF6 expression. Mechanistically, we demonstrated that MSI2-mediated stabilization of LINC00667 led to IRF6 mRNA degradation *via* the SMD pathway. This IRF6 reduction in turn relieved transcriptional repression of TJ-associated proteins (ZO-1, occludin, and claudin-5). Collectively, these events form a coherent pathway regulating BTB integrity, where modulating any component affects barrier permeability. Furthermore, individual or combined modulation through MSI2 knockdown, LINC00667 knockdown and IRF6 over-expression significantly enhanced Dox delivery across the BTB, potentiating its pro-apoptotic and anti-tumor effects in GB. These results not only clarify the molecular mechanisms governing BTB permeability but also offer a promising therapeutic strategy for improving drug delivery in GB treatment.

## Experimental procedures

### Cell lines and cell culture

The microvascular endothelial cell line hCMEC/D3 (ECs) was generously sponsored by Dr Couraud (Cochin Institute). ECs were grown in rat collagen I-coated (150 μg/ml; R&D Systems) Transwell inserts (0.4 μm pore size). ECs were cultured in EBM-2 medium containing 5% fetal bovine serum (SORFA) and the following additives: 5 mg/ml ascorbic acid (Sigma-Aldrich), 10 mM hepes, 1.4 mM hydrocortisone (Solarbio), 1% chemically defined lipid concentrate (Thermo Fisher Scientific), and 1 ng/ml human basic fibroblast growth factor (Sigma-Aldrich). The human glioma U251 and HEK293 T cell lines were acquired from the Shanghai Institutes for Biological Sciences Cell Resource Center. Both cell types were grown in DMEM containing 10% fetal bovine serum (TBD science). Sciencell Research Laboratories) provided the normal human astrocytes (NHAs). Poly-L-Lysine (sciencell) and astrocyte medium (sciencell) were used to cultivate NHAs. Standard culture conditions were maintained at 37 °C with 5% CO_2_ and appropriate humidity for all cell cultures.

### Establishment of BTB and BBB model *in vitro*

The establishment of BBB model *in vitro* was performed as follows. First, NHAs cells were plated in six-well plates at a density of 2 × 10^4^/well. Concurrently, ECs were cultured separately in the upper chamber of Transwell inserts at a seeding density of 2 × 10^5^ cells/well. When ECs reached nearly 50% confluency, the upper chamber of the Transwell containing ECs was carefully transferred to the six-well plate containing NHAs cells. Both ECs and NHAs were maintained in EBM-2 medium, with medium replacement every 48 h. Following 96 h of co-culture, the *in vitro* BBB model was successfully established. The ECs cultured under these conditions were designated as AECs.

The BTB model *in vitro* was established using an approach similar to the BBB model, with U251 glioma cells substituted for NHAs. In this system, ECs co-cultured with glioma cells were designated as GECs (glioma-co-cultured ECs).

### qRT-PCR

Total RNA was isolated from cells using TRIzol reagent (Life Technologies).

Reverse transcription and PCR amplification were carried out using One Step PrimeScript RT-PCR Kit (Takara Bio). The 7500 Fast RT-PCR system (Applied Biosystems) was utilized to quantify gene expression, with GAPDH serving as the endogenous reference gene. The relative gene expression was calculated by the 2^−ΔΔCt^ method. All primer sequences are provided in Table 1.

**Table 1:** Primers used for qRT-PCR

Gene	Sequence(5′–3′)
MSI2	F:GCCCCACCATGAGTTAGATTCCAAG
	R:GTGTTCGCAGATAACCCGCCTAC
LINC00667	F:CGCCTGTTCTCGCCAATCTCTATG
	R:CGTGATTCTGGGAGGTCCATTCAAC
IRF6	F:CTCATTGCCCACCAGAAAGGACAG
	R:GCGGACACTGCCACTATCAAAGG
GAPDH	F:GGACCTGACCTGCCGTCTAG
	R:TAGCCCAGGATGCCCTTGAG
ZO-1	F:GCGGATGGTGCTACAAGTGATG
	R:GCCTTCTGTGTCTGTGTCTTCATAG
Occludin	F:TTAACTTCGCCTGTGGATGACTTC
	R:TCTTGCTCTGTTCTCTTTGACCTTC
claudin-5	F:GCCTTCCTGGACCACAACATC
	R:TCAGAGCCAGCACCGAGTC

### Western blot

The procedures for performing Western blot assays were previously disclosed (51). ZO-1 (61–7300), occludin (71–1500), claudin-5 (35–2500) were all purchased from Thermo Fisher Scientific at a dilution ratio of 1:500. The dilutions for other primary antibodies were as follows: MSI2 (1:2000 dilution, 10770-1-AP, Proteintech), IRF6 (1:2000, A3209, ABclonal), STAU1 (1:2000, 14225-1-AP, Proteintech), GAPDH (1:10,000, 60,004-1-lg, Proteintech). HRP-conjugated AffiniPure goat anti-mouse (SA00001–1) and goat anti-rabbit (SA00001–2) secondary antibodies (Proteintech) were used at 1:10,000.

### Cell transfection

The knockdown (sh-MSI2, sh-LINC00667, sh-IRF6, sh-STAU1, sh-NC) and over-expression (pre-MSI2, pre-LINC00667, pre-IRF6, pre-NC) plasmids were custom-designed and synthesized by GenePharma, GeneChem, and Syngenbio, respectively. The target sequences are provided in Table 2.

**Table 2:** shRNA target sequences

Gene	Target Sequence(5′–3′)
MSI2 (−)	AGGCACAGAGGGTTTGGCTTT
LINC00667 (−)	CAGGTGCTCAAGTCATTTA
IRF6 (−)	CCAACCTGATTGAGAGACAAA
STAU1 (−)	GCCGCAGGGAGTTTGTGATGC

For transfection experiments, ECs were plated in 24-well plates (1 × 10^5^/well). Upon reaching 70% confluency, transfection was then performed using Opti-MEM I and Lipofectamine LTX & Plus reagents (Thermo Fisher Scientific). Briefly, 500 ng plasmid DNA was mixed with 100 μl Opti-MEM I and 0.5 μl Plus reagents in EP tube. After 15 min, 1.75 μl Lipofectamine LTX was added. Afterwards, the transfection mixture was added to ECs in 24-well plates for 48 h culture. After 4 weeks of selection with geneticin (G418, G8160, Solarbio) and/or puromycin (P8230, Solarbio), we established stably transfected cell lines: MSI2 knockdown [MSI2(−)] and over-expressed [MSI2(+)] ECs, LINC00667 knockdown [LINC00667(−)] and over-expressed [LINC00667(+)] ECs, IRF6 knockdown [IRF6(−)] and over-expressed [IRF6(+)] ECs, STAU1 knockdown [STAU1(−)] ECs. Western blot and qRT-PCR assays were used to assess the transfection effeciency (Fig. S1).

After co-culture with different stably transfected ECs, the BTB models *in vitro* were established using U251 cells.

### TEER measurement

TEER is an approach to determine BTB integrity and permeability by detecting electrical resistance across cell monolayers. The procedures for performing TEER tests were previously disclosed (52). The Millicell-ERS device (Millipore) was used to obtain the TEER values. TEER (Ω·cm^2^) = (sample resistant value - background resistant value) × the surface area of the transwell insert.

To validate the sensitivity and reliability of the TEER method for detecting BTB permeability, Cytochalasin D (Cyto D) was used as a positive control to assess its effect on TEER values in an *in vitro* BTB model (52). The experiment was divided into three groups: Cyto D (1 μM, in the upper chamber), Vehicle Control (0.1% DMSO), and the NC (fresh culture medium).

### HRP flux

Barrier properties of the BTB model *in vitro* were characterized through HRP flux assay. At 48 h post-model establishment, HRP (0.5 μM, Sigma-Aldrich) was introduced into the upper chamber of the Transwell system. After 1 h incubation, 15 μl cultured medium was collected from the lower chamber and transferred to a 96-well plate. Then, 200 μl tetramethylbenzidine (TMB, Solarbio) was added, followed by 30-min incubation in a dark place. Absorbance (optical density, OD) was measured by spectrophotometer, and HRP flux was calculated and expressed as pmol passed per cm^2^ surface area per hour (pmol/cm^2^·h^−1^).

To evaluate the sensitivity and reliability of the HRP flux assay for assessing BTB permeability, Cyto D as a positive control to determine its effect on HRP fluxes in an *in vitro* BTB model. The experiment was divided into three groups: Cyto D (1 μM Cyto D and 0.5 μM HRP, in the upper chamber), Vehicle Control (0.1% DMSO and 0.5 μM HRP), and the NC (and 0.5 μM HRP).

### Immunofluorescence

The procedures for performing immunofluorescence tests were previously disclosed (53).The cells were spread into the glass-bottom cell culture dishes (201,200, SORFA) at an appropriate concentration. Fluorescence imaging was subsequently performed using confocal microscopy to visualize cellular morphology and distribution.

### FISH

Red fluorescent-labeled LINC00667 probes and the FISH detection kit were provided by GenePharma. The FISH assay was performed using a red fluorescent-labeled LINC00667 sense probe as the NC, and all experimental procedures strictly adhered to the manufacturer's protocol.

### RIP

RIP experiments were conducted with a kit from BersinBio, adhering to the standard procedures. In short, after lysing the GECs, DNA was removed from the cell lysis samples. The samples were divided into three parts: IP, IgG, and Input. For the IP group, lysates were incubated with either MSI2- or STAU1-specific antibodies, whereas the IgG control group received nonspecific IgG. RNA-protein complexes were then isolated using protein A/G magnetic beads, followed by qRT-PCR to assess binding efficiency.

### RNA pull-down

The experimental processes were conducted with a commercial RPD Kit (Thermo Fisher Scientific), adhering to the standard procedures. In this experiment, a biotin-labeled anti-sense RNA probe complementary to LINC00667 served as the NC. In short, both the target LINC00667 probe and the anti-sense control probe were renatured to facilitate secondary structure formation. Subsequently, each probe was independently incubated with streptavidin-coated magnetic beads to form probe-bead complexes. After lysing the GECs, DNA was removed from the the cell lysis sample. Then the samples were divided into three parts: IP, RPD, and NC. The probe-bound beads were added to each sample to obtain the RNP complexes, and binding efficiency was ultimately determined by Western blot.

### Nascent RNA capture

The nascent RNAs was detected by using the Click-iT RNA Capture Kit (Thermo Fisher Scientific). Following the instructions, GECs were incubated with 0.2 mM five-ethynyluridine for 2 h to incorporate the label into newly transcribed RNA, followed by total RNA extraction using TRIzol. A 10 μg aliquot of RNA was subjected to click chemistry with biotin-azide for 30 min at room temperature under dark conditions. After ethanol precipitation, the biotinylated RNA was isolated using streptavidin-coated magnetic beads. The purified nascent RNA was quantitatively analyzed by qRT-PCR.

### RNA half-life assay

When GECs stretched by 70% in the petri dishes, the cells were maintained in media containing actinomycin D (1:1000) to prevent *de novo* RNA synthesis. Total RNA was isolated from GECs at sequential time points (0, 2, 4, 6, and 8 h), and the mRNA expression of LINC00667 and IRF6 was quantified by qRT-PCR. The half-life of each transcript was calculated as the time required for expression levels to reduce by 50%.

### Reporter vector construction and dual-luciferase reporter assays

A Dual Luciferase Reporter Assay System (Promega) was employed to detect the binding between LINC00667 and IRF6. The WT and binding site-mutated IRF6 sequences were inserted into the firefly luciferase reporter vector pGL3-Basic to construct the pGL3-Basic-IRF6-3′UTR-WT (IRF6-3′UTR-WT) and pGL3-Basic-IRF6-3′UTR-MUT (IRF6-3′UTR-MUT) vectors(Fig. S4*H*), respectively. pre-LINC00667 (LINC00667) and pre-NC (LINC00667-NC) plasmids were custom-designed and synthesized by GeneChem. Five experimental groups were established: Control group, IRF6-3′UTR-WT + LINC00667-NC group, IRF6-3′UTR-WT + LINC00667 group, RF6-3′UTR-MUT + LINC00667-NC group, and RF6-3′UTR-MUT + LINC00667 group. The procedure was performed as follows: According to the experimental groups, the aforementioned vectors, plasmids, and the internal control Renilla luciferase reporter vector phRL-TK were co-transfected into HEK293 T cells. After 24 to 48 h, cells were lysed with 1× PLB, and the lysates were collected. Subsequently, LAR II and Stop & Glo reagents were sequentially added, and firefly and Renilla luciferase activities were measured using a luminometer. The ratio of firefly to Renilla luciferase activity was calculated to assess the binding ability bwtween LINC00667 and IRF6.

To detect the relationship between IRF6 and the promoters of its target genes (ZO-1, occludin, and claudin-5), potential IRF6 binding sites in the promoter regions (−2000 to 0 bp) of ZO-1, occludin, and claudin-5 were predicted by JASPAR database. Then, the pEX3 empty vector and pEX3-IRF6 vectors (with and without predicted binding sites) were constructed. HEK293T cells were transfected with the recombinant plasmids in separate groups. After transfection, the Dual Luciferase Reporter System was performed to quantify promoter activity.

### Chromatin immunoprecipitation (ChIP)

The association of IRF6 with promoter regions of ZO-1, occludin, and claudin-5 was examined using chromatin immunoprecipitation with the Simple ChIP Enzymatic Chromatin IP Kit from Cell Signaling Technology. In a word, the ChIP assay was used to obtain pure DNA, which was then amplified by qRT-PCR. The amplified DNA products were carried out by the agarose gel electrophoresis. Table 3 lists all primer sequences used in this study.

**Table 3:** Primers used for chromatin immunoprecipitation (ChIP)

Gene	Sequence(5′-3′)
ZO-1 PCR1 (control)	F:TGGTGTTCCATGGATAAACTCT
	R:AAGGTAAAACTGTTGCCAACAA
ZO-1 PCR2	F:GGAAAGCTTTGGTGCACAGG
	R:CGGAAGGAAAAGCCGGGTAA
occludin PCR1 (control)	F:GACTTGTTTCGTGGCTCAGC
	R:ACCCCATGATTAGAAACTTGTTGAC
occludin PCR2	F:TCCCCAAAGGAGAAACAACCC
	R:GCCCATTAATAGTCACTCCCCA
claudin-5 PCR1 (control)	F:CATTGTTCCACTGCAGCCTG
	R:CTCTCCCTTCTCACCTGGCT
claudin-5 PCR2	F:CCGTGTCCCTTTGTCTCTCC
	R:GGCTTCCCAGACCTCTCAAT

### Apoptosis

U251 cell apoptosis was assessed by adding Dox to the Transwell upper chamber and analyzing the cells in the lower compartment. After two washes with PBS, U251 cells were digested using EDTA-free trypsin and resuspended in PBS. Following centrifugation (1000 rpm, 5 min), the cell pellets were collected. The supernatant was discarded and cells were washed three times with PBS. The 10X buffer (from the Apoptosis Detection Kit, DONJINDO) was diluted to 1X with PBS. Then, 500 μl 1X buffer, 5 μl FITC conjugate and 5 μl PI solution were added to the centrifuged cells. The cell suspension was cultured for 15 min in the dark. After filtration, cell apoptosis was analyzed using flow cytometry (BD Accuri C6).

The gating strategy was as follows: the primary cell population was first identified on an FSC-A/SSC-A dot plot to exclude debris. Single cells were then selected from this population using an FSC-A/FSC-H dot plot to exclude doublets. Apoptosis analysis was conducted on the single-cell population using an Annexin V-FITC/PI dot plot. The quadrants were defined as follows: lower left (Annexin V-/PI-) for viable cells, lower right (Annexin V+/PI-) for early apoptotic cells, and upper right (Annexin V+/PI+) for late apoptotic/necrotic cells (Fig. S3*A*).

### The relative flux of dox in BTB model *in vitr*o

Transwell chambers were used to establish the BTB model *in vitr*o. After that, the BTB models were treated with 40 μM Dox in the upper Transwell chambe. After 2 h of treatment, the medium from the lower chamber was collected into 96-well plates. The OD values were acquired using an INFINITE E PLEX Multi-functional Microplate Reader (Tecan Austria GmbH) at excitation and emission wavelengths of 495 nm and 575 nm, respectively.

### Statistical analysis

Statistical analyses were performed using GraphPad Prism (v8.0), with data expressed as mean ± SD. Dual comparisons employed Student's *t* test, while multi-group analyses utilized one-way ANOVA and two-way ANOVA. Significance thresholds were set at ∗*p* < 0.05 and ∗∗*p* < 0.01. Triplicate independent experiments were conducted.

## Data availability

No datasets were generated or analyzed during the current study.

## Supporting information

This article contains supporting information.

## Conflict of interest

The authors declare that they have no conflict of interest with the contents of this article.

## Figures and Tables

**Supplementary Fig. S4:**