# Supplementary material for: The RNA-binding protein MSI2 controls blood-tumor barrier permeability via LINC00667-Mediated IRF6 mRNA decay
Source: J Biol Chem. 2026 Jan 23;302(3):111208. doi: 10.1016/j.jbc.2026.111208 (PMC12930058; doi:10.1016/j.jbc.2026.111208)
Supplement: Supplemental material [file mmc1.docx]

**Supplementary Fig. S1 The transfected efficiency of MSI2、LINC00667、IRF6、STAU1.**

**A, B.** The transfected efficiency of MSI2 konckdown was detected by qRT-PCR and Western blot assays. Data represent mean ± SD (n = 3). ^**^*P* < 0.01 vs. MSI2(-)NC group. **C, D.** The transfected efficiency of MSI2 over-expression was detected by qRT-PCR and Western blot assays. Data represent mean ± SD (n = 3). ^**^*P* < 0.01 vs. MSI2(+)NC group. **E, F.** The transfected efficiency of LINC00667 konckdown and overexpression was detected by qRT-PCR asssays. Data represent mean ± SD (n = 3). ^**^*P* < 0.01 vs. LINC00667(-)NC group, ^**^*P* < 0.01 vs. LINC00667(+)NC group. **G, H.**

The transfected efficiency of IRF6 overexpression was detected by qRT-PCR and Western blot assays. Data represent mean ± SD (n = 3). ^**^*P* < 0.01 vs. IRF6(+)NC group. **I, J.** The transfected efficiency of IRF6 konckdown was detected by qRT-PCR and Western blot assays. Data represent mean ± SD (n = 3). ^**^*P* < 0.01 vs. IRF6(-)NC group. **K, L.** The transfected efficiency of STAU1 konckdown was detected by qRT-PCR and Western blot assays. Data represent mean ± SD (n = 3). ^**^*P* < 0.01 vs. STAU1(-)NC group.

**Supplementary Fig. S2 The positive control of TEER and HRP flux assay.**

**A, B.** Effect of cytochalasin D (Cyto D) (1 µM) on TEER and HRP measurement, Data represent mean ± SD (n = 3).

**Supplementary Fig. S3 The step-by-step gating process of apoptosis analysis and standard curve of Dox.**

1. The FSC-A/SSC-A dot plot was to exclude debris. The FSC-A/FSC-H dot plot was to exclude doublets. Q1-LL: Viable cells, Q1-LR: Early Apoptotic cells, Q1-UR: Late Apoptotic cells. Apoptotic cell (%) = Q1-LR + Q1-UR. **B.** The standard curve of Dox. The measurement Wavelength was 495nm. Data represent mean (n = 3).

**Supplementary Fig. S4 Bioinformatics database and software prediction results.**

1. Integrated analysis of catRAPID, GSE263588, GSE119834 three datasets yielded 179 lncRNAs that were highly expressed in glioma tissues/GSCs and could bind MSI2. **B.** The kockdown efficiency of MSI2 was detected by qRT-PCR. Data represent mean ± SD (n = 3). ^**^*P* < 0.01 vs. Control group. **C.** The binding of MSI2 and LINC00667 was predicted by RPISeq database. **D.** The LINC00667-binding sites in MSI2 was predicted by RNAInter. **E, F.** The Alu sequence in LINC00667 and IRF6 was predicted by RepeatMasker database. G**.** The LINC00667 and IRF6 can bind by Alu sequence was predicted by RNAInter database. **H.** Schematic diagrams of the firefly luciferase vectors containing the wild-type and mutant IRF6 sequences, along with the internal control renilla luciferase vector.
